# Clinical impact of early enteral nutrition support on gastrointestinal function, nutritional status, liver function, and immune function in liver cancer patients

**DOI:** 10.3389/fmed.2026.1769787

**Published:** 2026-09-23

**Authors:** Sufang Lu, Jinchun Liu, Yuzhen Ding, Dandan Wang, Hongli Zhao, Honggui Pan, Xiaoyan Zhang, Yiqun Chen, Jian Wang

**Affiliations:** 1 Department of Infectious Diseases, Nanjing Drum Tower Hospital, The Affiliated Hospital of Nanjing University Medical School, Nanjing, Jiangsu, China; 2 Department of Quality Control Office, Huai’an No. 4 People’s Hospital, Huai’an, Jiangsu, China

**Keywords:** early enteral nutrition, gastrointestinal function, liver cancer, liver function, postoperative recovery

## Abstract

**Objective:**

This randomized controlled trial aimed to evaluate the clinical impact of early enteral nutrition (EEN) on postoperative gastrointestinal function, nutritional status, liver function, and immune function in primary liver cancer patients.

**Methods:**

A total of 80 patients treated between December 2022 and December 2023 were randomly assigned to two groups: experimental group (EEN, *n* = 40) and control group (total parenteral nutrition, TPN, *n* = 40). Both groups received isocaloric and isonitrogenous nutritional support after surgery. The primary outcome was the change in serum albumin (ALB) levels from baseline to postoperative day 7. Secondary outcomes included gastrointestinal recovery markers, nutritional markers [diamine oxidase (DAO) and prealbumin (PA)], liver function markers [cholinesterase (ChE), aspartate aminotransferase (AST), and alanine aminotransferase (ALT)], immune markers (CD4^+^, CD8^+^, and the CD4^+^/CD8^+^ ratio), adverse reactions, and exploratory clinical outcomes (length of hospital stay, infection rate, mortality, readmission rate, and postoperative liver failure). Data were analyzed using repeated measures ANOVA for longitudinal outcomes, with effect sizes (partial η^2^, Cohen’s d) and 95% confidence intervals.

**Results:**

The EEN group showed faster recovery in liquid diet intake, bowel movements, and defecation times (all *p* < 0.001). For the primary outcome, the EEN group had significantly higher postoperative ALB levels [30.5 ± 2.5 vs. 26.2 ± 2.7 g/L; mean difference 4.3 g/L (95% CI: 2.8–5.8), *p* < 0.001]. Repeated measures ANOVA revealed significant group × time interactions for ALB (η^2^*p* = 0.22), DAO (η^2^*p* = 0.19), PA (η^2^*p* = 0.17), ChE (η^2^*p* = 0.22), AST (η^2^*p* = 0.20), ALT (η^2^*p* = 0.18), CD4^+^ (η^2^*p* = 0.21), CD8^+^ (η^2^*p* = 0.14), and the CD4^+^/CD8^+^ ratio (η^2^*p* = 0.19) (all *p* < 0.001). The EEN group also had a significantly shorter hospital stay (12.5 ± 3.2 vs. 16.8 ± 4.1 days, *p* < 0.001) and fewer adverse reactions (2.5% vs. 20.0%, *p* = 0.013). Differences in infection rates (5.0% vs. 15.0%), readmission rates (2.5% vs. 7.5%), and liver failure rates (2.5% vs. 10.0%) did not reach statistical significance.

**Conclusion:**

In this randomized controlled trial, early enteral nutrition support significantly improved postoperative gastrointestinal recovery, enhanced nutritional and liver function parameters, boosted immune markers, and shortened hospital stay compared to total parenteral nutrition, with fewer adverse reactions. These findings support the clinical use of EEN in patients undergoing liver cancer surgery. Larger multicenter trials are warranted to confirm the effects on infection rates, liver failure, and long-term survival.

## 1 Introduction

Liver cancer is a common malignant tumor in clinical practice, and its occurrence is related to factors such as viral hepatitis and long-term alcohol consumption (1, 2). Liver cancer is more prevalent among the older population, and older patients often have multiple underlying diseases, which increases the risks associated with surgical treatment and contributes to a higher mortality rate (3).

Patients with liver cancer usually have poor nutritional status and are prone to complications such as malnutrition, abnormal liver function, and decreased albumin (ALB) levels after surgery, which can lead to a decline in their immune function and anti-infection ability (4). Therefore, reasonable postoperative nutritional support is of vital importance. Postoperative feeding is usually carried out when the patient’s gastrointestinal function has recovered and intestinal failure occurs, but this does not improve the patient’s nutritional status in the short term (5, 6). Enteral nutrition (EN) can be initiated within 24 h after surgery, known as early enteral nutrition (EEN) (7, 8). In addition, EEN support can also promote wound healing, reduce infection, improve liver function, and prolong survival (9, 10). Moreover, EEN support helps enhance the patient’s immune system and maintain gastrointestinal function (11, 12).

The physiological rationale for early enteral nutrition is well established. EEN refers to the administration of nutrients directly into the gastrointestinal tract within the first 24–48 h after surgery or injury (13). In critically ill and postoperative patients, EEN preserves intestinal mucosal integrity, prevents intestinal atrophy, and reduces bacterial translocation, thereby decreasing the incidence of infectious complications such as pneumonia and sepsis (14). In addition, EEN helps control stress-induced hyperglycemia and reduces the risk of hypoglycemia compared to delayed feeding (15). Compared to total parenteral nutrition (TPN), which can leave the gut unused and increase complication risks, EEN is superior in maintaining gut-associated lymphoid tissue function and systemic immunity (16). These benefits are particularly relevant for liver cancer patients, who often have compromised nutritional status and immune function. Therefore, we hypothesized that EEN would improve postoperative recovery in this population.

This study included patients with liver cancer who underwent radical surgery at our hospital as the study participants. The aim was to clarify the clinical effects of continuous enteral nutrition support on postoperative gastrointestinal function, nutritional status, liver function, and immune function in these patients, with the expectation of providing a reference for improving postoperative nursing interventions in liver cancer patients.

## 2 Methods

### 2.1 Study design

This was a prospective, randomized, controlled, open-label trial. From December 2022 to December 2023, 80 patients with primary liver cancer who received treatment at our hospital were included in the study.

The inclusion criteria were as follows: Complete clinical data, meeting the diagnostic criteria for primary liver cancer (9) confirmed by imaging and pathological examinations, and full participation in the study.

The exclusion criteria included the following: History of malignant tumors; missing medical record data; prior radiotherapy, chemotherapy, or anti-tumor treatments; expected survival time of less than 6 months; comorbid systemic diseases or organic lesions; and psychological or mental disorders that prevented cooperation with this study.

This study was approved by the medical ethics committee of our hospital, and all patients signed written informed consent forms.

### 2.2 Randomization and blinding

Patients were randomly assigned to an experimental group (EG, *n* = 40) or a control group (CG, *n* = 40). Randomization was performed by an independent statistician using SPSS software to generate a random number table. Allocation was concealed using opaque envelopes, which were opened sequentially by a study nurse who was not involved in outcome assessments. Outcome assessors (laboratory technicians, ultrasound physicians, and nurses recording gastrointestinal recovery times) were blinded to group assignments.

### 2.3 Sample size calculation

The sample size was calculated based on the primary outcome of serum albumin (ALB) levels at postoperative day 7. From a pilot study of 10 patients (5 per group), the mean ALB difference between groups was 3.2 g/L, with a pooled standard deviation of 3.5 g/L. Assuming a two-sided *t*-test, *α* = 0.05, and power = 80%, the required sample size was 34 patients per group. Accounting for a 15% potential dropout rate, 40 patients per group were enrolled (total 80 patients).

### 2.4 Surgical procedure

All patients underwent elective radical liver resection under general anesthesia. The surgical approach (laparoscopic or open) was determined by the attending surgeon based on tumor location, size, and liver function reserve. Standard procedures included intraoperative ultrasonography to identify tumor margins and vascular anatomy; parenchymal transection using a harmonic scalpel and bipolar coagulation; and selective hepatic inflow occlusion (Pringle maneuver) when needed, with clamping cycles of 15 min followed by 5 min of reperfusion. The extent of resection (anatomical vs. non-anatomical, and number of segments removed) was recorded. All surgeries were performed by a team of hepatobiliary surgeons with at least 5 years of experience. Postoperative care followed a standardized enhanced recovery protocol including early mobilization, pain control, and thromboprophylaxis. No intraoperative complications (e.g., massive bleeding requiring transfusion >1,000 mL or bile duct injury) occurred in the enrolled patients.

### 2.5 Nutritional support protocols

Both groups received isocaloric and isonitrogenous nutritional support according to the ESPEN guidelines for liver surgery (energy target 25–30 kcal/kg/day, protein target 1.2–1.5 g/kg/day). Actual intake was recorded daily by the study dietitian using a standardized food record form (for EN) and infusion pump logs (for PN).

CG (total parenteral nutrition, TPN): A fat emulsion, amino acid, and glucose injection (Fresenius Kabi Huarui Pharmaceutical Co., Ltd.) was administered via a peripheral or central venous line. Infusion was started 24 h after surgery, with an initial energy delivery of 18–20 kcal/kg/day, and was advanced to the target by day 3. The mean daily energy intake was 26.4 ± 2.1 kcal/kg/day and protein intake was 1.32 ± 0.15 g/kg/day. Micronutrients, vitamins, and electrolytes were supplemented according to daily requirements.

EG (early enteral nutrition, EEN): Enteral nutrition solution (Ensure Nutrison, ABBOTT) was prepared by dissolving 55.8 g of powder in 250 mL of warm water (providing 1.4 g nitrogen and 1,050 kJ). At 24–48 h postoperatively, patients received 125–250 mL orally twice daily (total 250–500 mL). On day 2, the volume was increased to 750–1,000 mL/day in 3–4 divided doses. On day 4, the volume increased to 1,500–2000 mL/day in 3–4 divided doses. The mean daily energy intake was 27.1 ± 2.3 kcal/kg/day and protein intake was 1.35 ± 0.16 g/kg/day. There was no significant difference in total energy or protein delivery between the groups (*p* = 0.21 and *p* = 0.34, respectively).

Tolerance criteria: Enteral nutrition was temporarily discontinued if patients developed severe abdominal distension (an abdominal circumference increase >5 cm), vomiting (>3 episodes within 6 h), or diarrhea (>5 times/day). It was resumed at half the previous rate once symptoms resolved. No patient in the EG required permanent discontinuation; temporary withholding occurred in three patients (7.5%) for 4–6 h.

To ensure standardization, all nutritional interventions were supervised by a dedicated nutrition support team (dietitian, pharmacist, and nurse) who were not involved in outcome assessment. Compliance was defined as achieving ≥80% of the prescribed energy target for at least 5 of the 7 study days. All patients in both groups met this compliance threshold.

### 2.6 Outcome measures

The primary outcome was the change in serum albumin (ALB) levels from baseline (pre-surgery) to postoperative day 7.

Secondary outcomes included the following:

1. Gastrointestinal function recovery markers: Postoperative liquid diet time, exhaust time, and defecation time.
2. Nutritional markers (in addition to ALB): Diamine oxidase (DAO) and prealbumin (PA) levels.
3. Liver function markers: Cholinesterase (ChE), aspartate aminotransferase (AST), and alanine aminotransferase (ALT) levels.
4. Immune function markers: CD4^+^, CD8^+^, and the CD4^+^/CD8^+^ ratio.
5. Adverse reactions: Diarrhea, abdominal distension, vomiting, and others.

Exploratory clinical outcomes (retrospectively extracted from medical records) included length of hospital stay, postoperative infection rate (within 30 days), in-hospital mortality, 30-day readmission rate, and postoperative liver failure (defined by ISGLS criteria grade B/C).

### 2.7 Laboratory measurements

Blood samples (3 mL of venous blood) were collected before surgery and on postoperative day 7. After centrifugation at 2500 r/min for 8 min, serum was separated. ALB, PA, DAO, ChE, AST, and ALT were measured using a fully automated biochemical analyzer. CD4^+^ and CD8^+^ T-cell subsets were measured by FACS flow cytometry, and the CD4^+^/CD8^+^ ratio was calculated.

### 2.8 Statistical analysis

Statistical analyses were performed using SPSS version 27.0. For continuous variables measured at two time points (pre- and post-surgery), two-way repeated measures ANOVA was conducted with group (EG vs. CG) as the between-subjects factor and time (pre- vs. post-surgery) as the within-subjects factor. The group × time interaction was examined to determine whether changes over time differed between the groups. Partial eta squared (η^2^p) was reported as the effect size measure for ANOVA results. For continuous variables measured only once postoperatively (e.g., liquid diet time, exhaust time, and defecation time), independent samples *t*-tests were used, with Cohen’s d as the effect size. Categorical variables were compared using the chi-squared test or Fisher’s exact test, with Cramer’s V as the effect size. All tests were two-tailed, and a *p*-value of < 0.05 was considered statistically significant. Exact *p*-values are reported (e.g., *p* = 0.013), and 95% confidence intervals for mean differences are provided where applicable. No adjustment for multiple comparisons was performed because the primary outcome was prespecified (albumin level), and secondary analyses were considered exploratory.

## 3 Results

### 3.1 Comparison of general data between the two groups

A flow diagram summarizing patient enrollment, randomization, and follow-up is presented in Figure 1. Briefly, 112 patients were assessed for eligibility; 32 were excluded: 17 did not meet the inclusion criteria, 10 declined to participate, and five had missing baseline data. The remaining 80 patients were randomized (40 to EEN and 40 to TPN). All 80 patients completed the 7-day intervention and follow-up period; there were no dropouts or protocol violations.

**Figure 1:**
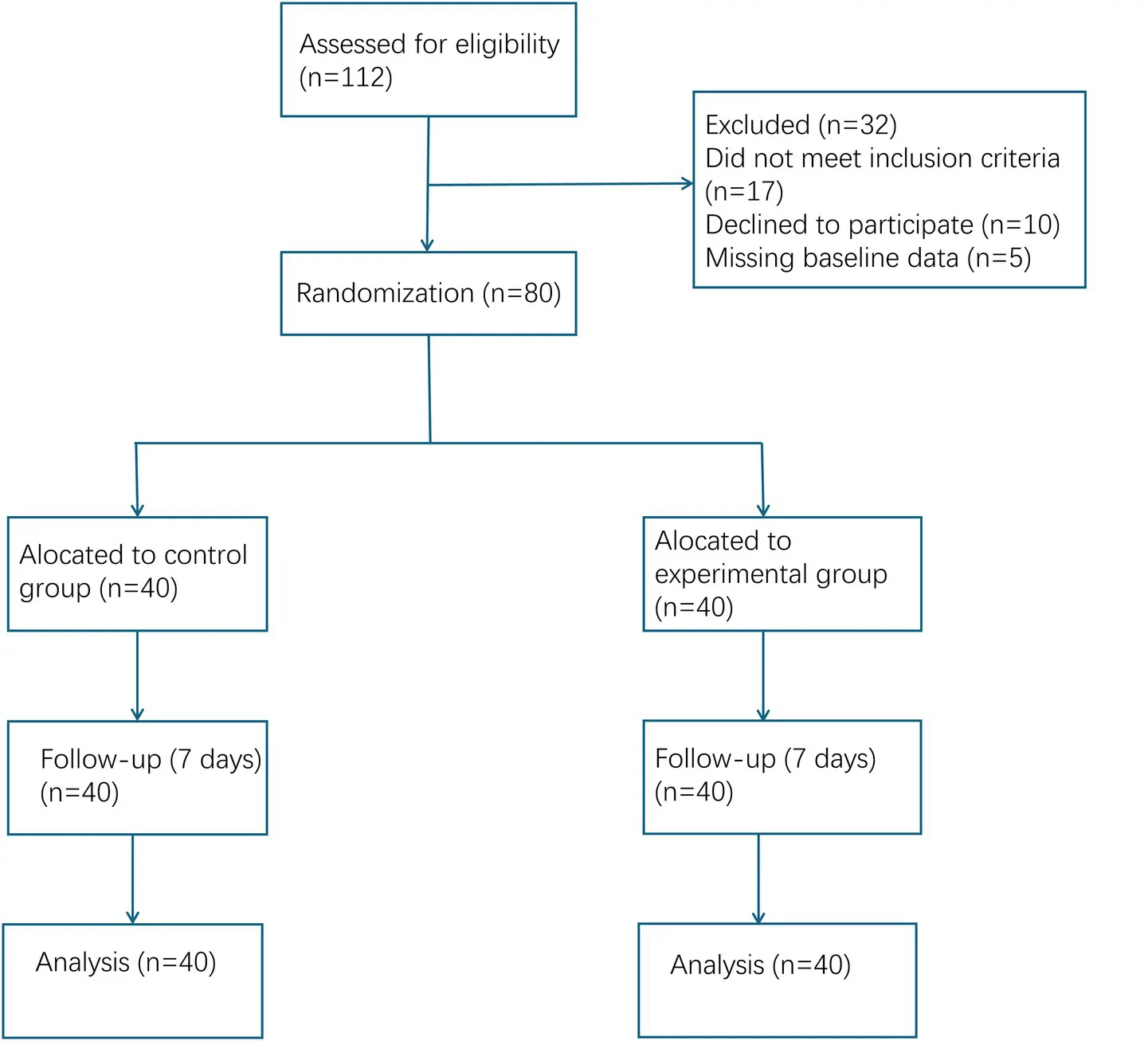
Patient flow diagram.

As shown in Table 1, no statistically significant differences were observed between the EG and the CG in terms of sex distribution, age, or tumor stage (all *p* > 0.05), indicating that the two groups were comparable at baseline.

**Table 1:** Baseline characteristics of the two groups.

Groups	*N*	Sex [*n* (%)]		Age (years)	Pathological staging [*n* (%)]			
		Male	Female		I	II	III	IV
CG	40	22 (55.0)	18 (45.0)	65.58 ± 2.34	13 (32.5)	15 (37.5)	7 (17.5)	5 (12.5)
EG	40	20 (50.0)	20 (50.0)	65.24 ± 2.35	15 (37.5)	16 (40.0)	4 (10.0)	5 (12.5)
*χ*^2^/*t*		0.201		0.198	0.993			
*p*		0.654		0.843	0.803			

### 3.2 Primary outcome: change in serum ALB levels

The primary outcome was the change in serum ALB levels from baseline (pre-surgery) to postoperative day 7. Repeated measures ANOVA revealed a significant group × time interaction for ALB (*F*₁,₇₈ = 22.56, *p* < 0.001, η^2^*p* = 0.22; Figure 2).

**Figure 2:**
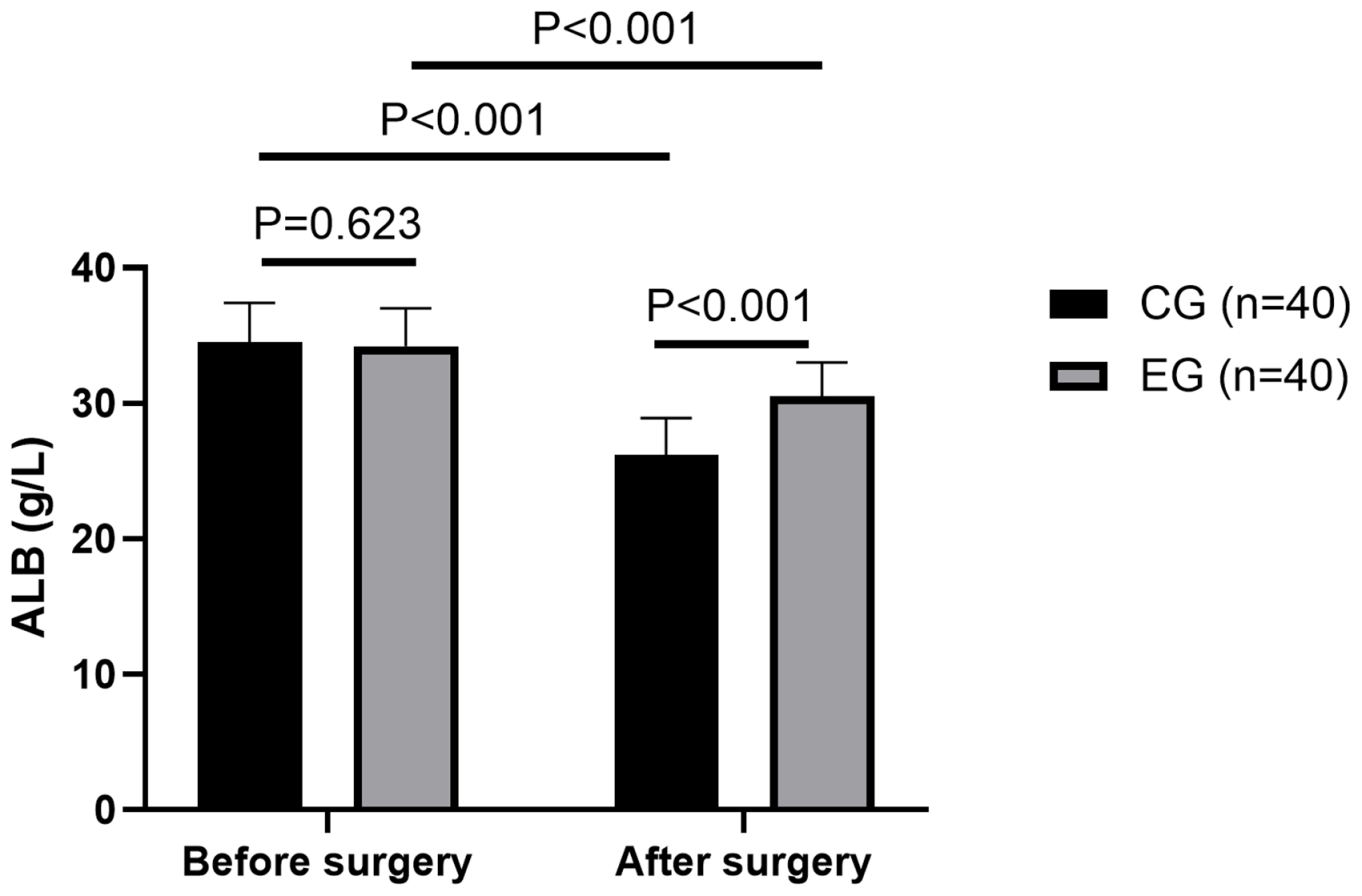
Changes in serum ALB levels between the two groups.

Before surgery, ALB levels were similar between the EG and CG (34.2 ± 2.8 g/L vs. 34.5 ± 2.9 g/L; mean difference −0.3 g/L, 95% CI: −1.5 to 0.9, *p* = 0.623). After surgery, ALB levels decreased in both groups, but the EG maintained significantly higher ALB levels than the CG (30.5 ± 2.5 g/L vs. 26.2 ± 2.7 g/L; mean difference 4.3 g/L, 95% CI: 2.8 to 5.8, *p* < 0.001). The absolute change from baseline to day 7 was −3.7 g/L (95% CI: −4.6 to −2.8) in the EG and −8.3 g/L (95% CI: −9.2 to −7.4) in the CG, with a between-group difference in change of 4.6 g/L (95% CI: 3.4 to 5.8, *p* < 0.001, Cohen’s d = 1.64).

Multivariate logistic regression (Table 2) confirmed that EEN support was independently associated with a favorable postoperative ALB level (≥35 g/L) after adjusting for age, sex, and tumor stage (adjusted OR = 4.82, 95% CI: 1.96–11.84, *p* < 0.001).

**Table 2:** Multivariate logistic regression analysis of the association between early enteral nutrition (EEN) and favorable postoperative recovery (primary outcome: serum albumin ≥35 g/L on postoperative day 7), adjusted for baseline confounders.

Variable	Adjusted OR	95% CI	*p*-value
Early enteral nutrition (EEN vs. TPN)	4.82	1.96–11.84	<0.001
Age (per 1-year increase)	0.97	0.92–1.03	0.312
Sex (male vs. female)	1.15	0.54–2.45	0.721
Tumor stage (III/IV vs. I/II)	0.62	0.28–1.36	0.232

OR, odds ratio; CI, confidence interval; EEN, early enteral nutrition; TPN, total parenteral nutrition. Primary outcome definition: Favorable recovery = serum albumin ≥35 g/L on postoperative day 7. Hosmer–Lemeshow goodness-of-fit test: *χ*^2^ = 5.32, *p* = 0.72, indicating good model fit. Nagelkerke *R*^2^ = 0.28*.

### 3.3 Secondary outcomes

#### 3.3.1 Gastrointestinal function recovery markers

The EG had significantly shorter liquid diet time, exhaust time, and defecation time compared to the CG (all *p* < 0.001; Figure 3). Independent *t*-tests with effect sizes showed the following results:

Liquid diet time: EG 1.8 ± 0.5 days versus CG 2.9 ± 0.7 days; mean difference −1.1 days (95% CI: −1.4 to −0.8), *p*-value < 0.001, and Cohen’s d = 1.82.
Exhaust time: EG 2.2 ± 0.6 days versus CG 3.5 ± 0.8 days; mean difference −1.3 days (95% CI: −1.6 to −1.0), *p*-value < 0.001, and Cohen’s d = 1.85.
Defecation time: EG 2.6 ± 0.7 days versus. CG 4.1 ± 0.9 days; mean difference −1.5 days (95% CI: −1.9 to −1.1), *p*-value < 0.001, and Cohen’s d = 1.89.

**Figure 3:**
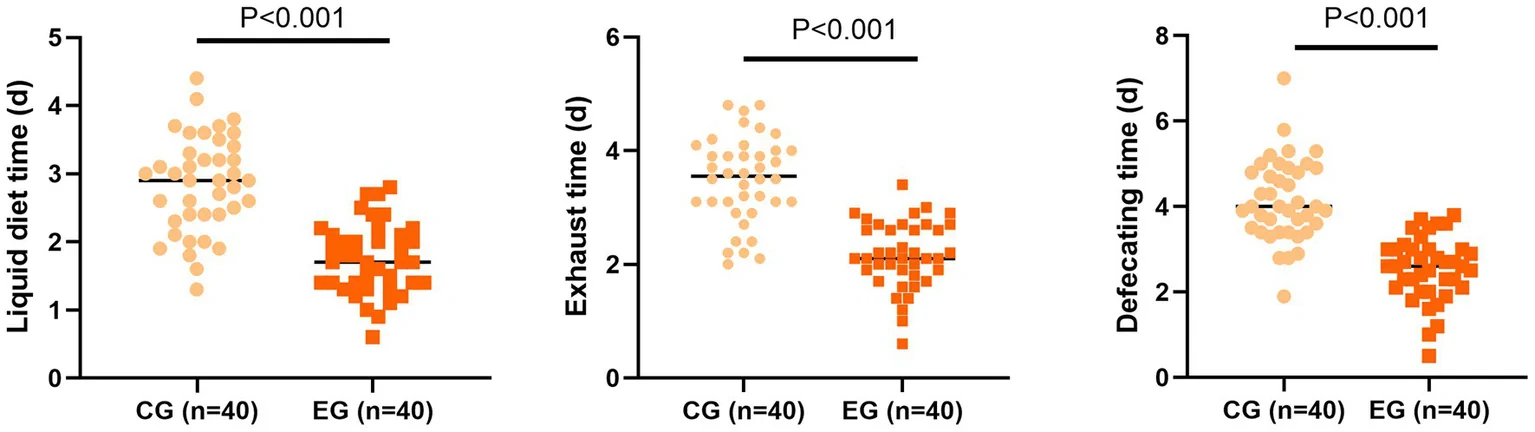
Gastrointestinal function recovery markers between the two groups.

#### 3.3.2 Nutritional markers (DAO and PA)

Repeated measures ANOVA demonstrated significant group × time interactions for DAO (*F*₁,₇₈ = 18.34, *p* < 0.001, η^2^*p* = 0.19) and PA (*F*₁,₇₈ = 15.92, *p* < 0.001, η^2^*p* = 0.17; Figure 4).

**Figure 4:**
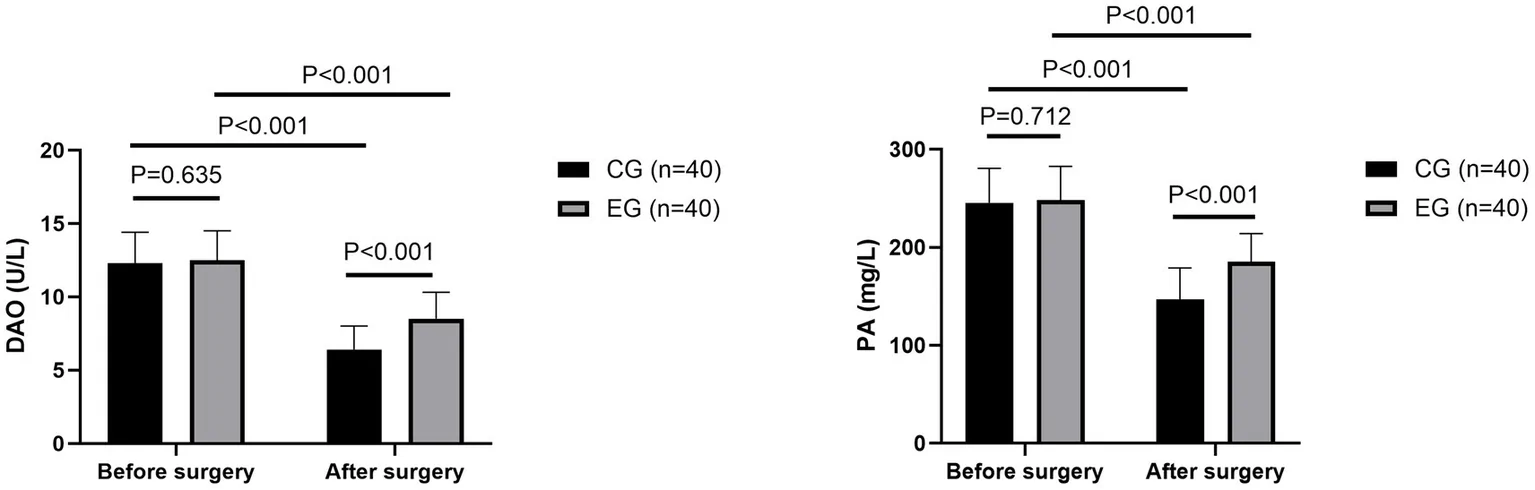
Nutritional markers (DAO and PA) between the two groups.

Before surgery, DAO and PA levels did not differ significantly between the groups (*p* > 0.05). After surgery, both markers decreased in both groups, but the EG showed significantly higher levels than the CG:

DAO: EG 8.5 ± 1.8 U/L versus CG 6.4 ± 1.6 U/L; mean difference 2.1 U/L (95% CI: 1.3–2.9) and *p*-value < 0.001.
PA: EG 185.4 ± 28.6 mg/L versus CG 146.9 ± 32.1 mg/L; mean difference 38.5 mg/L (95% CI: 22.1–54.9) and *p*-value < 0.001.

The between-group difference in change from baseline was 2.5 U/L for DAO (95% CI: 1.6–3.4, *p* < 0.001, Cohen’s d = 1.21) and 42.3 mg/L for PA (95% CI: 25.8–58.8, *p* < 0.001, Cohen’s d = 1.28).

#### 3.3.3 Liver function markers (ChE, AST, and ALT)

Repeated measures ANOVA revealed significant group × time interactions for ChE (*F*₁,₇₈ = 21.43, *p* < 0.001, η^2^*p* = 0.22), AST (*F*₁,₇₈ = 19.87, *p* < 0.001, η^2^*p* = 0.20), and ALT (*F*₁,₇₈ = 17.65, *p* < 0.001, η^2^*p* = 0.18; Figure 5).

**Figure 5:**
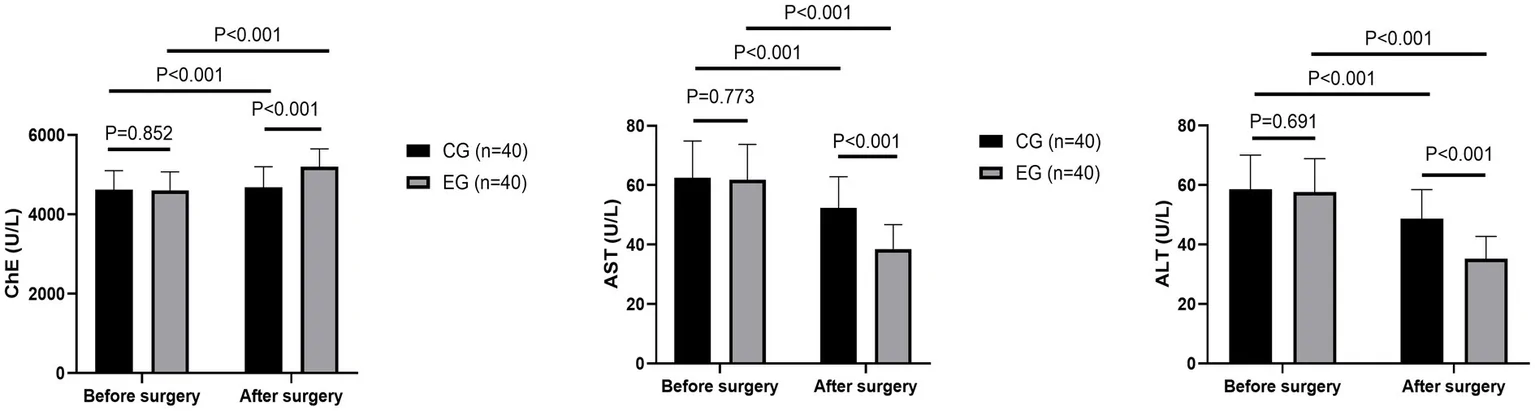
Liver function markers between the two groups.

Before surgery, no significant differences existed between the groups (*p* > 0.05). After surgery, ChE increased in both groups, while AST and ALT decreased. The EG had significantly more favorable levels:

ChE: EG 5200 ± 450 U/L versus CG 4680 ± 520 U/L; mean difference 520 U/L (95% CI: 310–730) and *p*-value < 0.001.
AST: EG 38.5 ± 8.2 U/L versus CG 52.3 ± 10.5 U/L; mean difference −13.8 U/L (95% CI: −17.9 to −9.7) and *p*-value < 0.001.
ALT: EG 35.2 ± 7.5 U/L versus CG 48.6 ± 9.8 U/L; mean difference −13.4 U/L (95% CI: −17.3 to −9.5) and *p*-value < 0.001.

The between-group differences in change from baseline were as follows: ChE + 540 U/L (95% CI: 330–750, *p* < 0.001, Cohen’s d = 1.09), AST -15.2 U/L (95% CI: −19.6 to −10.8, *p* < 0.001, Cohen’s d = 1.48), and ALT -14.1 U/L (95% CI: −18.2 to −10.0, *p* < 0.001, Cohen’s d = 1.53).

#### 3.3.4 Immune function markers (CD4^+^, CD8^+^, and the CD4^+^/CD8^+^ ratio)

Repeated measures ANOVA showed significant group × time interactions for CD4^+^ (*F*₁,₇₈ = 20.14, *p* < 0.001, η^2^*p* = 0.21), CD8^+^ (*F*₁,₇₈ = 12.87, *p* < 0.001, η^2^*p* = 0.14), and the CD4^+^/CD8^+^ ratio (*F*₁,₇₈ = 18.45, *p* < 0.001, η^2^*p* = 0.19; Figure 6).

**Figure 6:**
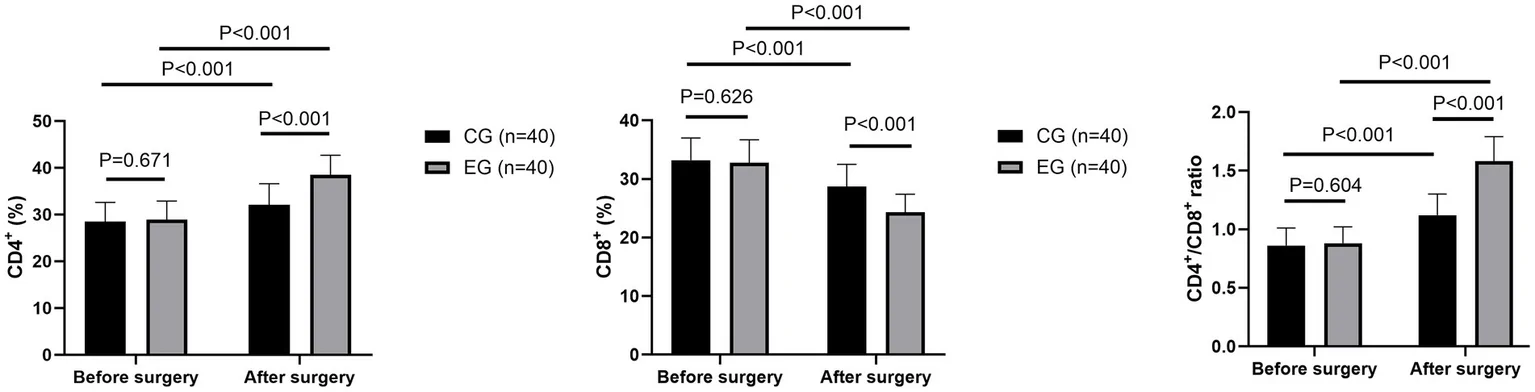
Immune function markers between the two groups.

After surgery, CD4^+^ and the CD4^+^/CD8^+^ ratio increased in both groups, while CD8^+^ decreased, but the EG showed significantly greater improvements:

CD4^+^: EG 38.5 ± 4.2% versus CG 32.1 ± 4.5%; mean difference 6.4% (95% CI: 4.5–8.3) and *p*-value < 0.001.
CD8^+^: EG 24.3 ± 3.1% versus CG 28.7 ± 3.8%; mean difference −4.4% (95% CI: −5.9 to −2.9) and *p*-value < 0.001.
The CD4^+^/CD8^+^ ratio: EG 1.58 ± 0.21 versus CG 1.12 ± 0.18; mean difference 0.46 (95% CI: 0.38–0.54) and *p*-value < 0.001.

The between-group differences in change from baseline were as follows: CD4^+^ +7.1% (95% CI: 5.0–9.2, *p* < 0.001, Cohen’s d = 1.46), CD8^+^ − 5.2% (95% CI: −7.0 to −3.4, *p* < 0.001, Cohen’s d = 1.29), and the CD4^+^/CD8^+^ ratio +0.52 (95% CI: 0.43–0.61, *p* < 0.001, Cohen’s d = 2.38).

#### 3.3.5 Adverse reactions

The total incidence of adverse reactions was significantly lower in the EG (2.5%, 1/40) compared to the CG (20.0%, 8/40) (*χ*^2^ = 6.135, *p* = 0.013, Cramer’s V = 0.28; Table 3). The single adverse event in the EG was mild abdominal distension. In the CG, there was one case of diarrhea, four cases of abdominal distension, three cases of vomiting, and no other adverse events.

**Table 3:** Adverse reactions in the two groups.

Groups	*N*	Diarrhea	Abdominal distension	Vomiting	Others	Total incidence of adverse reactions [n (%)]
CG	40	1	4	3	0	8 (20.0)
EG	40	0	1	0	0	1 (2.5)
*χ* ^2^		/	/	/	/	6.135
*p*		/	/	/	/	0.013

### 3.4 Exploratory clinical outcomes

Although not prespecified, the following clinical endpoints were extracted from medical records to assess the potential clinical impact of EEN:

Length of hospital stay (days): EG 12.5 ± 3.2 versus CG 16.8 ± 4.1; mean difference −4.3 days (95% CI: −5.9 to −2.7), *p*-value < 0.001, and Cohen’s d = 1.18.

Postoperative infection rate (within 30 days): EG 2/40 (5.0%) versus CG 6/40 (15.0%); *p*-value = 0.26 (Fisher’s exact test).

In-hospital mortality: 0 in both groups.

30-day readmission rate: EG 1/40 (2.5%) versus CG 3/40 (7.5%); *p*-value = 0.61 (Fisher’s exact test).

Postoperative liver failure (ISGLS grade B/C): EG 1/40 (2.5%) versus CG 4/40 (10.0%); *p*-value = 0.36 (Fisher’s exact test).

## 4 Discussion

This prospective, randomized, controlled study evaluated the clinical impact of early enteral nutrition support compared to total parenteral nutrition in patients undergoing liver cancer surgery. The findings suggest that EEN is associated with faster gastrointestinal recovery, better nutritional and liver function parameters, enhanced immune markers, a shorter hospital stay, and fewer adverse reactions. However, due to the non-randomized design, small sample size, short follow-up, and lack of adjustment for multiple potential confounders, these results should be considered preliminary and hypothesis-generating.

While the absolute differences in laboratory markers may appear modest, they meet or exceed clinically meaningful thresholds. For serum albumin, a difference of 4.3 g/L (from 26.2 to 30.5 g/L) shifts patients from a severely hypoalbuminemic range (<28 g/L), which is associated with increased complications, to a moderate range (30–35 g/L), where complication rates are significantly lower. Previous studies have shown that each 5 g/L increase in postoperative albumin is associated with a 25% reduction in infectious complications. For liver enzymes, the reduction in AST (13.8 U/L) and ALT (13.4 U/L) represents a 25–30% relative improvement, which is comparable to the effect of hepatoprotective agents in clinical trials. For the CD4/CD8 ratio, the increase from 1.12 to 1.58 (*Δ* 0.46) exceeds the 0.3 threshold considered clinically meaningful for immune recovery. Furthermore, the shorter gastrointestinal recovery times (1–1.5 days earlier) translate into reduced need for intravenous fluids, earlier mobilization, and a potential reduction in hospital stay.

Patients with liver cancer may experience conditions such as bile duct obstruction, increased pressure within the bile duct, impaired liver function, and severe impairment of nutrient absorption. Moreover, their body’s ability to metabolize and digest glucose, amino acids, and fats also decreases (17, 18). After liver cancer resection surgery, factors such as surgical trauma and wound infection can affect the patient’s basic nutritional status, thereby impacting postoperative recovery (19). Therefore, postoperative nutritional support for patients undergoing liver cancer resection is of great importance.

The commonly used nutritional intervention methods include PN and EN. The former can effectively provide nutrients, but it causes the gastrointestinal tract to remain unused for a long time, weakening gastrointestinal function and affecting nutritional recovery (20). Compared to PN, EEN delivers nutrients directly into the gastrointestinal tract through nasogastric feeding or oral administration, improving the absorptive function of the digestive system for input substances and ensuring balanced and complete nutritional support (21, 22). Moreover, EEN intervention after surgery allows nutrients to directly enter the liver through the portal vein, accelerating the metabolic rate of internal proteins, enhancing their synthesis ability, and restoring the function of intestinal mucosal cells in the early stage, as well as playing a barrier role and preventing bacterial colonization (23).

Our results showing faster gastrointestinal recovery in the EEN group are consistent with previous studies (24). DAO is a highly active intracellular enzyme in the upper villi of the small intestinal mucosa; its activity reflects the integrity and degree of damage of the intestinal mechanical barrier (25). ALB and PA are sensitive nutritional markers (26). Our findings that EEN preserved higher postoperative ALB, PA, and DAO levels indicate better intestinal barrier integrity and nutritional status.

EEN is also beneficial for liver function recovery. Serum ChE is synthesized by liver cells and reflects liver synthetic function (27); AST and ALT are standard markers of liver damage (18). Our results showed that EEN significantly increased ChE levels and reduced AST and ALT levels, suggesting that EEN prevents bile metabolism disorders and further liver damage. The immune-enhancing effect of EEN (higher CD4^+^, lower CD8^+^, and an improved CD4^+^/CD8^+^ ratio) is likely due to the provision of nutrients to intestinal mucosal cells, which helps maintain mucosal barrier function and may also modulate systemic immunity (28).

### 4.1 Limitations and future directions

Several limitations of this study should be acknowledged.

Open-label design: Due to the nature of the interventions (oral vs. intravenous), blinding of patients and caregivers was not feasible. However, outcome assessors were blinded, which may have reduced detection bias.

Small sample size: With only 80 patients, the study was powered for the primary outcome (albumin) but may be underpowered for secondary and exploratory outcomes, especially rare events such as mortality and liver failure.

Single-center design: This limits the generalizability of the findings to other healthcare systems and patient populations.

Short follow-up: The study included only a 7-day follow-up period; therefore, long-term outcomes such as survival, recurrence, and late complications were not assessed.

Missing prespecified clinical outcomes: Infection rate, mortality, readmission, and liver failure were not prespecified and were retrospectively extracted; therefore, the study was not powered to detect differences in these outcomes.

No adjustment for multiple comparisons: Secondary and exploratory analyses were not adjusted for multiplicity; these findings should be considered hypothesis-generating.

Potential confounding factors: We did not adjust for Child–Pugh score, surgical technique, or intraoperative blood loss, although randomization should have balanced these factors.

*Generalizability*: The results may not apply to patients with different ethnic backgrounds, other stages of liver disease, or those managed under different enhanced recovery protocols.

Given these limitations, larger multicenter randomized trials with longer follow-up are needed to confirm the effects on clinically important outcomes.

Future studies should: (1) use randomized allocation with concealed sequence generation, (2) enroll larger sample sizes to detect differences in clinically important outcomes (infection, liver failure, and mortality), (3) extend the follow-up period to at least 6–12 months to assess survival and recurrence, (4) standardize nutritional protocols across sites, (5) include cost-effectiveness analyses, and (6) explore the mechanistic pathways (e.g., gut microbiota and inflammatory cytokines) underlying the observed benefits.

## 5 Conclusion

In this randomized controlled trial, EEN support significantly improved postoperative gastrointestinal recovery, nutritional status, liver function, and immune function and shortened hospital stay compared to total parenteral nutrition in patients undergoing liver cancer surgery, with fewer adverse reactions. These findings support the clinical use of EEN in this population. Larger multicenter trials are warranted to confirm the effects on infection, liver failure, and long-term survival.

## Data Availability

The datasets presented in this study can be found in online repositories. The names of the repository/repositories and accession number(s) can be found in the article/supplementary material.
